# La Manœuvre de tous les Accouchemens contre Nature, reduite à sa plus grande Simplicité

**Published:** 1833-10

**Authors:** 


*La Manœuvre de tous les Accouchemens contre Nature, reduite à sa plus grande Simplicité, et précédée du Mécanisme de l'Accouchement naturel.* Par JULES HATIN, Docteur en Médecine de la Faculté de Paris. *Seconde Edition.* Paris, 1832. Pp. 369.

THIS does not appear to us to be a very important work; and, though the author imagines that he has filled up an aching void in obstetric literature, we are sure that the student who is in possession of Denman and Merriman need never refer to Dr. Hatin. He says, at page 242, speaking of face presentations,

"In positions of this kind the delivery must never be left to nature, although, in some rare cases, it may be accomplished by the unassisted efforts of the woman; for the labour is so long and difficult, that the life of the child is almost always sacrificed."

Dr. Hatin accordingly recommends either turning, or the application of the forceps. Not so Merriman, who holds (and rightly, we think,) that, in general, the best plan is to let these cases alone:

"I am quite convinced that, as a general rule, it is wrong to turn in face cases; yet, under some very favourable circumstances, such an operation may perhaps be admissible. If, for instance, the head of the child were found lying above the brim of the pelvis, the membranes unruptured, the os uteri well dilated, and the vagina and perineum in a state of relaxation, the probability of saving the life of the child would be considerable, and might justify the operator in having recourse to such a proceeding.

"Professor Bang, in the *Acta Nov. Reg. Soc. Medic. Havn.*, vol. i., 1818, relates ten cases of face presentation.

"In 3 cases the child was turned, and in every case was stillborn.

"In 4 cases, after ineffectual attempts to turn, the children were at length expelled by the pains, and were all stillborn.

"In one case the forceps was used about twenty-four hours after the labour began: the child was dead.

"In 2 cases, left entirely to nature, the children were born alive.

"All the mothers recovered.

"In most of the cases, turning seems to have been undertaken sooner than would allow the parts to be properly dilated. I have twice known the presentation of the face converted by the pains alone into a natural presentation." (*Merriman on Difficult Parturition*, 4th edition, p. 47-8.)

Dr. Hatin is gratified to think that his book, being a pocket volume, can be carried about by the accoucheur, and consulted whenever he is puzzled. Quere: Is this ever done; and are Vade-mecums allowed to verify their names? We think not.

